# Effects of two novel denture cleansers on multispecies microbial biofilms, stain removal and the denture surface: an in vitro study

**DOI:** 10.1186/s12903-023-03535-5

**Published:** 2023-11-11

**Authors:** Rattiporn Kaypetch, Pachara Rudrakanjana, Peerapong Tua-ngam, Orada Tosrisawatkasem, Sarut Thairat, Pairin Tonput, Pornpen Tantivitayakul

**Affiliations:** 1 Department of Oral Microbiology, Faculty of Dentistry, Mahidol University, 6 Yothi Street, Rajthevi, Bangkok, 10400 Thailand; 2 Research office, Faculty of Dentistry, Mahidol University, Bangkok, Thailand

**Keywords:** Antimicrobial effects, Multispecies microbial biofilm, Novel denture cleansers, Stain removal, Physical properties

## Abstract

**Background:**

The continuously increasing demand for removable denture appliances and the importance of adequate denture cleaning have led to the development of various denture cleansing products. The aim of this study was to evaluate the efficacy of two novel denture cleansing agents (GE and TM) and three commonly available cleansers (0.5% sodium hypochlorite; NaClO, 0.12% chlorhexidine gluconate; CHX, and Polident®; POL) on multispecies microbial biofilm formation, stain removal and physical properties of dentures.

**Methods:**

The antimicrobial activities of denture cleansing agents were determined against major oral opportunistic pathogens including *Streptococcus mutans, Staphylococcus aureus, Escherichia coli* and *Candida albicans*, using time-kill assays. Multispecies microbial biofilms grown on acrylic resins for 72 h were generated to determine the antibiofilm effects of cleansing agents by confocal laser scanning microscopy (CLSM). Evaluations of the tea and coffee stain removal properties and the alterations in the physical properties of dentures were also performed. The toxicity of cleanser residues released from denture acrylics to fibroblast cells was investigated using MTT assay.

**Results:**

All denture cleansing agents tested could effectively kill oral bacteria and *Candida albicans.* Furthermore, after immersion for more than 3 h, the cleansers Polident®, GE and TM could efficiently penetrate and inhibit multispecies denture biofilms with effects similar to 10 min of immersion in 0.5% NaClO. However, immersion in 0.12% CHX for 20 min showed less antibiofilm activity. The NaClO solution had the highest efficacy for removing stains from the artificial teeth. Conversely, the CHX solution enhanced tea and coffee staining, and the teeth immersed in this solution showed clinically unacceptable colour changes (ΔE > 5.5). However, the colour differences of teeth stained and immersed in POL, GE and TM cleansers were in the clinically acceptable range. There was no significant difference among the POL, GE and TM cleansers in terms of stain removal efficacy. The cleansers GE and TM did not alter the surface roughness and colour of the materials, moreover the residues of both cleansers did not exhibit cytotoxicity.

**Conclusion:**

Two novel denture cleansing agents containing natural products, GE and TM exhibited effective antimicrobial activity, antibiofilm and stain removal capabilities without toxicity or disturbance of the physical properties of acrylics.

**Supplementary Information:**

The online version contains supplementary material available at 10.1186/s12903-023-03535-5.

## Introduction

The shift in the distribution of the world’s population towards older ages is known as population ageing. The World Health Organization (WHO) estimated that 1 billion people were aged 60 years or older in 2019 and this number is expected to increase twofold in 2050 [1]. There has been an increase in the demand for removable denture appliances among the elderly population. Denture appliances can restore and improve four oral functions: mastication, swallowing, aesthetics and phonetic components [2]. Proper and regular denture care is an essential requirement for the use of removable dentures; however, it is a very common problem for elderly denture wearers who cannot adequately brush the dentures [3] due to diseases related to brain disorders and poor manual dexterity.

Denture appliances provide a surface for microbial adhesion and lead to the formation of multispecies microbial biofilms. Denture biofilms serve as a reservoir for various pathogenic microbes including *Candida* spp., *Streptococcus mutans*, *Staphylococcus aureus* and *Escherichia coli* [4–6]. Recent studies have reported that the presence of pathogens in denture plaque is associated with local infection (denture stomatitis and dental caries) and systemic infections such as aspirate pneumonia and gastrointestinal infection [4, 7]. Therefore, the crucial proper cleaning of denture appliances could prevent microbial plaque formation and reduce the risk of denture stomatitis, dental caries, periodontal disease and halitosis [8–10].

There are two main denture cleaning methods, mechanical and chemical [11, 12]. Mechanical cleaning includes the use of toothbrushes and ultrasonic devices. The commercially available denture cleaning chemicals can be categorized according to their chemical composition: bleach-based (sodium hypochlorite), peroxide-effervescent type and enzyme-based denture cleansers [11]. In recent years, nanoparticles [13], antimicrobial peptides [14] and plant extracts [15] have received significant attention as antimicrobial agents for dental applications. The use of water and a toothbrush is the most common method for denture cleaning [16] due to the low cost, simplicity and effectiveness. However, this method is abrasive to dentures and produces scratches when brushing with a hard bristled brush. This leads to high risks of microbial colonization and plaque formation on dentures [17]. The efficacy of the disinfection protocols of chemical denture cleansers has been widely studied; for example, 10 min of immersion in 0.5% NaClO solution could eliminate microbes without significant changes in the colour and surface roughness of dentures [18] and the immersion in 0.12% CHX for 20 min can eliminate Candida biofilms from dentures [19, 20]. For Polident®, the manufacturer’s recommended immersion time is 15 min. In this study, two novel chemical denture cleansers containing natural products were developed, geraniol (GE) and thymol (TM), which have potent antimicrobial and antibiofilm effects. The aim of this study was to evaluate the efficacy of 5 different cleansers on the removal of multispecies microbial biofilms, tea and coffee stains and determine their effect on the physical properties of dentures.

## Materials and methods

### Preparation of denture cleansing solutions

Five denture cleansing agents were used as the experimental agents as shown in Table S1. The denture cleansing solutions were freshly prepared prior to performing the experiments. Briefly, 1 tablet of the Polident®, Geraniol (GE) and Thymol (TM) denture cleansers were dissolved in 150 mL of sterile distilled water.

### Microbial strains and growth conditions

Reference strains of *Streptococcus mutans* ATCC 25175, *Streptococcus sanguinis* ATCC 10556, *Staphylococcus aureus* ATCC 6538, *Escherichia coli* ATCC 25922 and *Candida albicans* ATCC 10231 were used in this study. Bacterial strains were cultured in brain heart infusion (BHI) agar (Difco, Detroit, USA) in a CO_2_ incubator at 37 °C for 24 h. *Candida* spp. were grown in sabouraud dextrose agar (SDA; Difco, Detroit, USA) in an incubator at 37 °C for 48 h. The cultures were used in further experiments.

### Time-kill assays

Time kill assays with the *S. mutans*, *S. aureus*, *E. coli*, and *C. albicans* reference strains were performed according to a study of Klepser et al. [21] with some modifications. Briefly, 2–3 bacterial colonies were grown in BHI broth in a CO_2_ incubator overnight at 37 °C and then adjusted to a concentration of 1 × 10^8^ CFU/mL. A colony of *C. albicans* was grown in yeast nitrogen broth (YNB) medium with 2% glucose at 37 °C for 24 h. *Candida* inoculums were prepared at a concentration of 1 × 10^7^ CFU/mL. Next, 1 mL of bacterial or *Candida* cultures were aliquoted in 15 mL tubes containing 9 mL of medium (growth control) or cleansing solutions (ratio of 1:10) including Polident®, 0.12% CHX, 0.5% NaClO, GE solution or TM solution. Then these tubes were incubated for 5, 15, 30 min, 1 and 3 h. After incubation for different time periods, these tubes were centrifuged, the solutions were discarded and 1 mL of fresh medium was added to each. Finally, 100 µL of bacterial or Candida suspensions was serially diluted tenfold in 0.9% normal saline (NSS) and plated onto BHI agar or SDA agar, respectively. The number of viable microbial colonies was counted after 48 h of incubation. The killing activity was defined as a greater than 3log10-fold reduction in colony forming units per milliliter, or a 99.9% decrease in the initial inoculum. The experiments were conducted in triplicate.

### Preparation of denture acrylic specimens

Two shapes of denture acrylic specimens were used in this study: *i*) a disk-shaped pattern 10 mm in diameter and 2 mm thick for biofilm assays and *ii*) a rectangular pattern with dimensions of 15 mm × 15 mm × 2 mm for evaluating the physical properties and cytotoxicity assay. The acrylic specimens were prepared from a polymer based on methyl methacrylate (Vertex Heat-Curing Acrylic powder, Vertex-dental BV). The resins were manipulated, packed and pressed into a stainless-steel mold according to the manufacturer’s instructions. Heat polymerization was performed in water at 100 °C for 20 min and then allowed to cool to room temperature. The rectangular specimens were polished with abrasive paper grits of 400, 600, 800 and 1000, while the circular specimens were finished with 400- and 600-grit sandpaper. All acrylics were immersed in distilled water at room temperature for 3 d to eliminate any residual monomers. Finally, the acrylics were immersed in 70% ethanol for 10 min for disinfection and washed with sterile water for 10 min.

### Multispecies biofilm development on denture acrylics

Prior to developing the microbial biofilms on the denture acrylics, the acrylics were incubated in unstimulated saliva from four healthy researchers for 2 h in an incubator at 37 °C. The saliva was pooled and centrifuged at 5,000 × g for 10 min at 4 °C. Then, the saliva supernatant was filtered through 0.2 μm syringe filter and stored at -80 °C until use.

Five acrylics were placed in each well of a 6-well plate. The microbial inoculums were prepared by adding equal volumes (1.5 mL) of each microbial culture (*S. sanguinis, S. mutans, S. aureus, E. coli* and *C. albicans*) into each well containing the denture acrylics. The plates were incubated in a shaking incubator at 35 rpm overnight to allow the microbes to attach to the acrylic samples. Next, the strips were washed with PBS to remove the unattached cells and 8 mL of BHI medium with 2% sucrose was added. Then, the plate was incubated in a CO_2_ incubator at 37 °C for 3 days to form mature biofilms. The medium was carefully changed every 24 h.

### Evaluation of the inhibitory effects on preformed multispecies microbial biofilms grown on denture acrylics

Seventy-two acrylic specimens with multispecies biofilm were immersed in 6 different solutions for different time intervals: *i*) 0.12% CHX for 20 min; *ii*) 0.5% NaClO for 10 min; *iii*) Polident® for 30 min, 3 and 6 h; *iv*) GE solution for 30 min, 3 and 6 h; *v*) TM solution for 30 min, 3 and 6 h and *vi*) media without test solutions (control group). After exposure to the cleansing solutions, the acrylic samples were rinsed three times with 0.9% NSS.

### Confocal laser scanning microscopy (CLSM)

The cell viability within the multispecies biofilms was measured by a staining kit (LIVE/DEAD BacLight, Molecular Probes, Eugene, OR, USA). The kit contains the fluorescent dyes SYTO-9 to identify live microbes with intact cell membranes, and propidium iodide (PI) to observe microbes with damaged cell membranes. Stock solutions of SYTO-9 and PI were diluted 1,000-fold in saline solution, and the acrylics were stained for 30 min at room temperature. After incubation with the dyes, the acrylic discs were placed on glass slides and visualized using a scanning fluorescence microscope (Leica, Dmi8, Leica Microsystems, Wetzlar, Germany) at 63 × magnification and detection at 488 and 568 nm for SYTO-9 and PI, respectively. Images of live and dead microbes within the biofilms were taken in the green and red channels or in the merged mode. Four random areas were selected and captured for each specimen. Three-dimensional sections of the biofilm in each area were obtained from image stacks (XY direction) taken at 2 μm intervals beginning at the bottom of the biofilm that was in contact with the acrylic surface to the top of the biofilm (Z-direction). The percentages of the viable and dead microbes were determined using colour-appropriated fluorescence intensity.

### Stain removal

The specimens consisted of artificial teeth, maxillary central incisors; shade A2 (Shofu Inc. Kyoto, Japan). Artificial teeth were divided into six groups (n = 10 per group) according to the types of denture cleansers. Before staining, the artificial teeth were immersed in artificial saliva (KCl 1.2 g/l, NaCl 0.84 g/l, CaCl_2_ 0.15 g/l, MgCl_2_ 0.05 g/l, K_2_HPO_4_ 0.34 g/l and mucin 3 g/l) [22] to form a pellicle layer and enhance the uptake of the stain.

The staining solutions used in this study including instant coffee (Nescafe Classic) and tea (Lipton Yellow Label Tea, Unilever, Turkey) were freshly prepared as follows. The coffee solution was prepared by adding 2 g of coffee to 100 mL of boiled water for 15 min. The tea solution was prepared by immersing one teabag into 100 mL of boiled water for 2 min. Both solutions were filtered through double layer gauze.

After the specimens were incubated with artificial saliva for 2 min and rinsed with water, they were immersed in the staining solutions for 14 h to simulate the weekly exposure time (2 h for 7 days) to these beverage [23]. After staining, the specimens were removed and rinsed with distilled water and air-dried. Afterwards, ten specimens were immersed in one of the five denture cleansers or distilled water for 7 h to simulate weekly cleansing (1 h for 7 days). Then they were rinsed with distilled water and air-dried. Therefore, the abovementioned steps were considered a weekly cleansing procedure, and this procedure was repeated 12 and 24 times to simulate the 3- and 6-months of cleanser usage.

Colour measurements were performed before staining (baseline), after staining and at the end of the 12^th^ and 24^th^ cleansing cycles. All specimens were washed with water, air-dried and then photographed with a clinical compact spectrophotometer (VITA Easyshade Compact, Germany). Specimens were placed on a white background and the probe tip was placed perpendicularly in the middle of each specimen. The measurements of the colour difference (ΔE) were performed by the L^*^a^*^b^*^ method under D65 standard lighting conditions in accordance with the CIELab system. (ΔE) was derived from the following equation: ΔE = [(ΔL)^2^ + (Δa)^2^ +(Δb)^2^]^½^. A ΔE value > 5.5 was considered clinically unacceptable and a ΔE value > 2.6 was considered as perceptible to the human eye [24].

### Cytotoxicity test

Evaluation of the cytotoxic effects of chemical residues impregnated into the acrylic samples immersed in the GE and TM cleansers was performed according to previous studies [25] with some modifications. Briefly, the acrylics (n = 6) were immersed in each cleansing solution for 3 h and then rinsed with water. The immersion cycle was repeated 180 times, simulating 6 months of daily cleansing. To prepare extracts from the specimens, the acrylic resins from each group were washed with distilled water to remove excess cleansers. Then, the specimens were placed in tubes containing 4.5 mL of tissue culture medium with a surface-to-fluid volume ratio of 6 cm^2^/mL. The specimens were incubated at 37 °C for 24 h to allow diffusion of the chemicals into the medium. Then the medium was sterilized by filtration through a 0.2 μm filter and kept at -20 °C until used. Extracts from polyurethane films containing 0.1% zinc diethyldithiocarbamate (PU-ZDEC, Hatano Research Institute, Tokyo, Japan) were used as a positive control. Furthermore, the cytotoxic effects of the resin components were determined using specimens without treatment (acrylic resin control).

The effects of the GE and TM cleansers released by the acrylic resins on the mouse areolar fibroblast cell line L929 (ATCC® Lot. No. 70026472, VA, USA) was adapted from ISO 10993-5:2009 [26]. L929 cells were cultured in minimum essential medium (MEM, Gibco, NY, USA) supplemented with 1% antibiotic–antimycotic (Gibco, NY, USA). Then the cells were plated into 96-well plates at a seeding density of 1 × 10^5^ cells/mL and incubated at 37 °C. One hundred microliters of extracted solutions were then added, and the cells were incubated for 24 h. Wells containing fresh culture medium without samples served as negative controls. Cell viability was assessed by using the MTT assay (Sigma-Aldrich, MO, USA). The absorbance was measured at 570 nm using a microplate spectrophotometer (BioTek Synergy H1 MFD). A reduction in cell viability by more than 30% was considered a cytotoxic effect.

### Immersion procedures for evaluation of the physical properties

One hundred eighty heat-cured acrylic resins were divided into 6 groups for physical property determination. Thirty specimens in each group were immersed in 100 mL of water or experimental solutions. The immersion time for each cycle is shown in Table S1. All specimens were rinsed with water after completing each cycle, and this procedure was continued 180 times to simulate 6 months of daily cleansing (T180). All specimens were kept in water when not immersed in test solution.

### Surface roughness

The surface roughness of the acrylics (n = 90; 15 specimens in each group) was evaluated using a surface roughness tester (Talysurf series 2, Taylor Hobson Ltd., Leicester, England). The stylus tip radius was 2 μm with a measuring length of 8 mm, a cut-off length of 0.8 mm, and a speed of 0.5 mm/s. The stylus was moved perpendicular to the direction of surface polishing and three measurements were made per specimen surface. The mean of the three recordings was then calculated. The roughness was measured before and after immersion at T180.

### Colour stability

The colour of the acrylic resins (n = 90; 15 specimens for each group) was measured before and after immersion at T180 using a colorimeter (ColorFlex 45/0, HunterLab, Reston, VA, USA). Colour was assessed by CIELAB colour space with three components of lightness: white-black (L), red-green (a), and yellow-blue (b). Therefore, three values were measured for each specimen and the mean values were recorded.

### Statistical analysis

Statistical analysis was performed using PASW Statistics 18 (SPSS Inc., Chicago, IL, USA) and GraphPad Prism software v.5.0 (GraphPad Software Inc., CA, USA). Difference in the killing effects of the microbial cells in the biofilms and stain removal, surface roughness and acrylic colour change data was analyzed among the experimental groups by using one-way ANOVA followed by Tukey’s post hoc test. A value of *p* < 0.05 was considered to indicate statistical significance.

## Results

### Time-kill assays

**Fig. 1:**
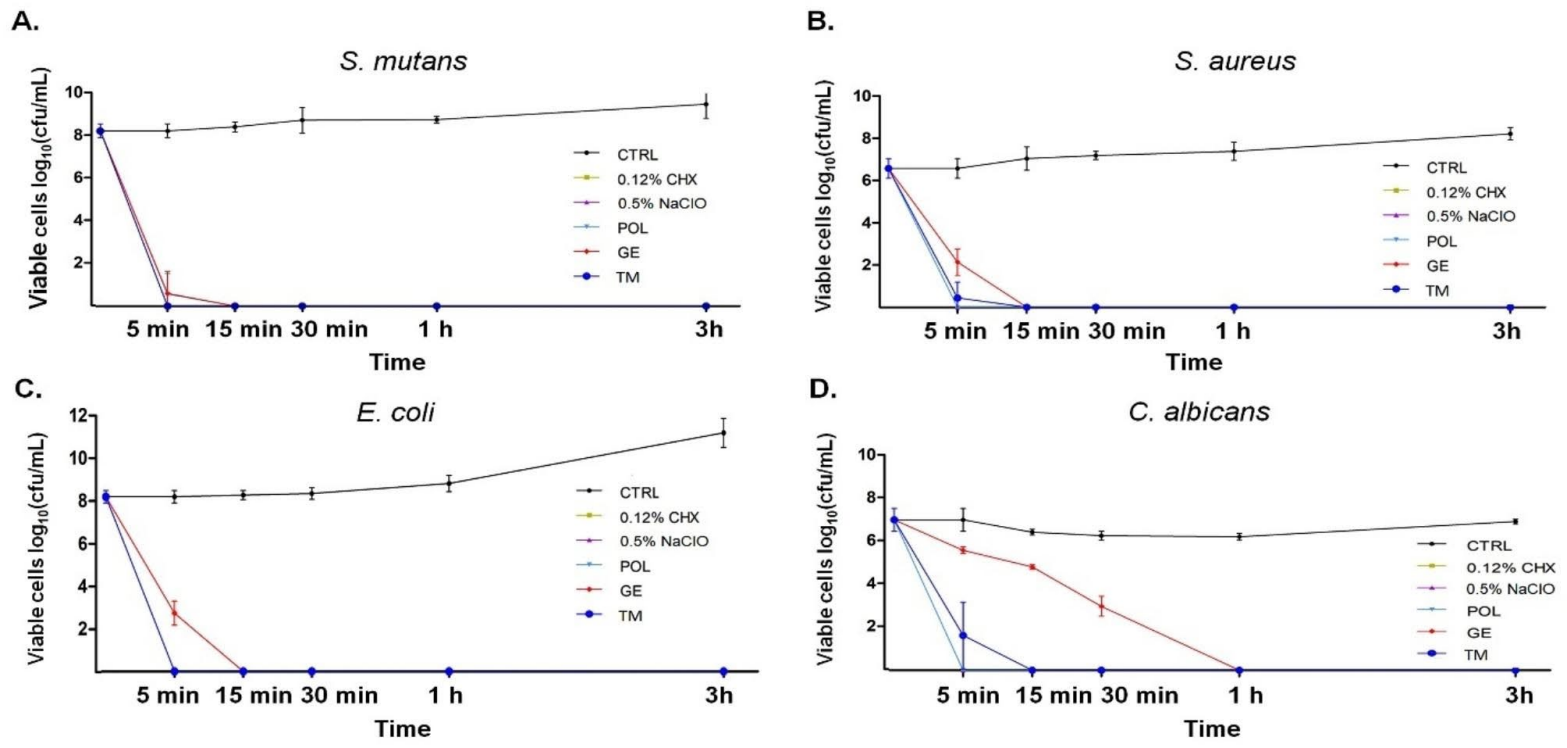
Time-kill curves of *S. mutans***(A)**, *S. aureus***(B)**, *E. coli***(C)** and *C. albicans***(D)** treated with 0.12% chlorhexidine (CHX), 0.5% sodium hypochlorite (NaClO), Polident® (POL), geraniol (GE) and thymol (TM) cleansing agents compared to the control group (medium without treatment; CTRL). Each symbol and error bar represent the mean and SD of log_10_CFU/mL of three independent experiments

Time kill assays were performed to determine the bactericidal and fungicidal effects of 5 different cleansing agents (0.5% NaClO, 0.12% CHX, POL, GE and TM) and compared them with the untreated control. The results showed that all cleansing solutions had more than 99.9% antibacterial activity after 5 min of treatment (Fig. 1A-C). Furthermore, all solutions except GE exhibited an anti-Candida effect of more than 99.9% after 5 min of incubation with the solutions. The GE solutions required approximately 30 min to kill 99.9% of *Candida* spp (Fig. 1D). Therefore, all cleansing agents could kill all tested bacterial genera belonging to gram-positive and gram-negative bacteria as well as *Candida* spp.

### Killing effects on the microbial cells of preformed multispecies biofilms grown on denture acrylics

**Fig. 2:**
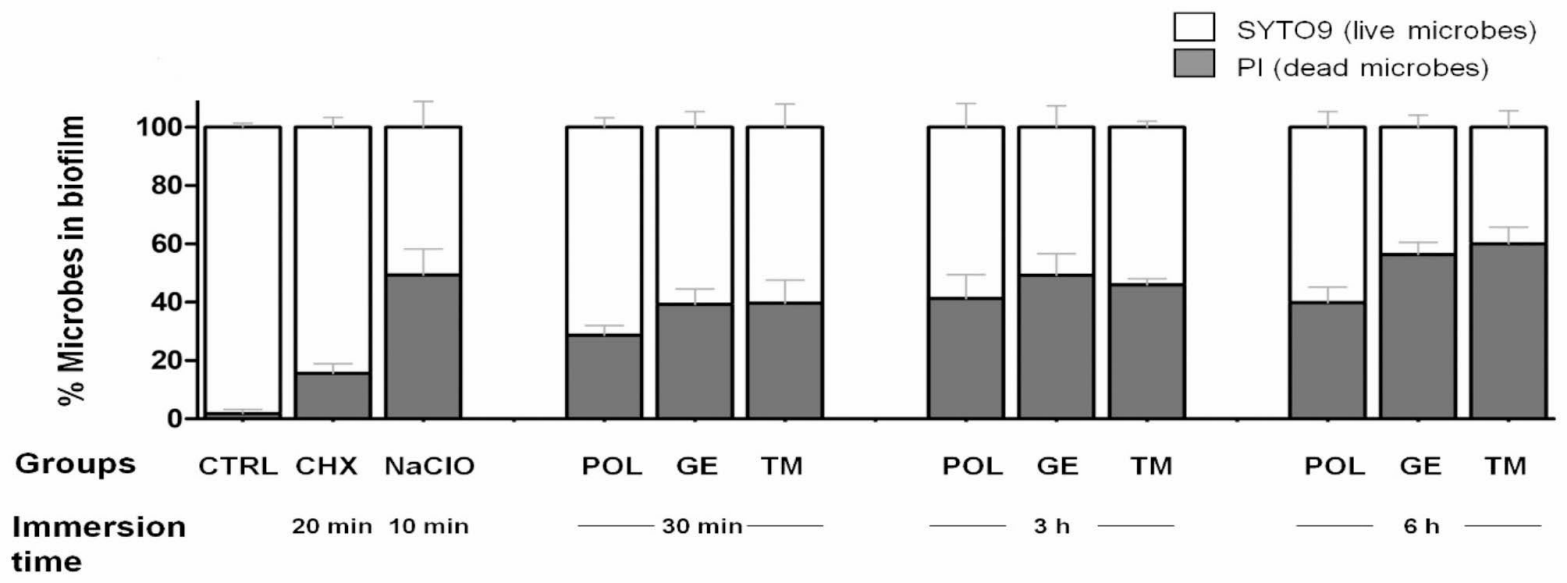
Assessment of the killing effect of 5 different cleansing solutions on microbial cells in 72-h-old multispecies biofilms compared to the control without treatment after different immersion times (n = 6 pieces per group). The percentages of nonviable (grey column) and viable (white column) microbes relative to total number of cells in the biofilm was calculated by dividing the red (PI) or green (SYTO9) fluorescence intensities by the total (green + red) fluorescence intensity of all sections from the bottom to top surface of the biofilms

**Fig. 3:**
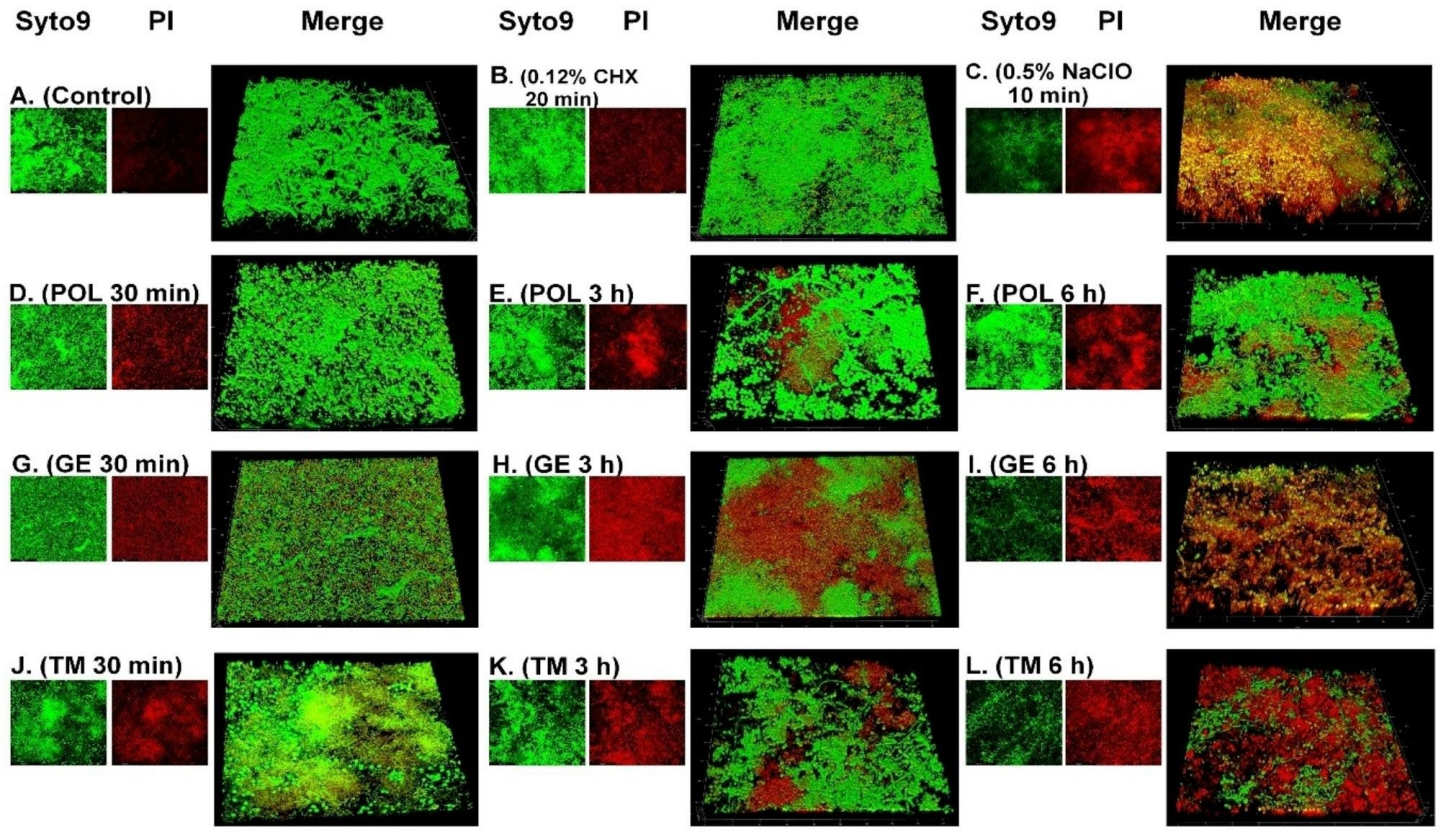
Confocal microscopy images of 72-h-old microbial biofilms stained with SYTO9 (green, live cells) and PI (red, dead cells) and the combined images showing all of the microbes within the biofilm after immersion in medium without treatment **(A)** and various cleansing solutions for 30 min, 3 and 6 h including CHX, 20 min **(B)**; NaClO, 10 min **(C)**; and POL **(D-F)**, GE **(G-I)**, and TM **(J-L)**.

The efficacy of the 5 different cleansers to remove the bacterial and candida cells within 72-h old biofilms was determined using confocal laser scanning microscopy and staining with a Live/Dead BacLight kit. The percentage of killed microbes in the biofilm following immersion in solutions was calculated by dividing the PI fluorescence intensity by the total fluorescence intensity (both SYTO9 and PI) of all sections from the bottom to top surface of the biofilm as shown in Fig. 2. The killing activity of 0.12% CHX after 20 min of immersion was significantly lower than that of the other treatment groups (*p* < 0.01) while, 0.5% NaClO for 10 min of immersion had significantly higher killing activity than 20 min of CHX and 30 min of POL treatment (*p* < 0.01). Nevertheless, 0.5% NaClO immersion had a killing effect comparable to that of immersion in POL, GE and TM for 3 and 6 h (*p* > 0.05) (Table S2). Furthermore, increasing the immersion time in POL, GE and TM solutions to 6 h significantly enhanced the killing of microbial cells in biofilms compared to 30 min of immersion (Table S2). The 3D CLSM biofilm images displayed increased red fluorescence after immersion in the POL, GE and TM cleansers for 3 and 6 h compared to those incubated for 30 min (Fig. 3D-L). Furthermore, immersion in the POL, GE, and TM cleansers for 6 h could not eradicate all *Candida* cells, seen as yeast and hyphal form in Fig. 3.

**Fig. 4:**
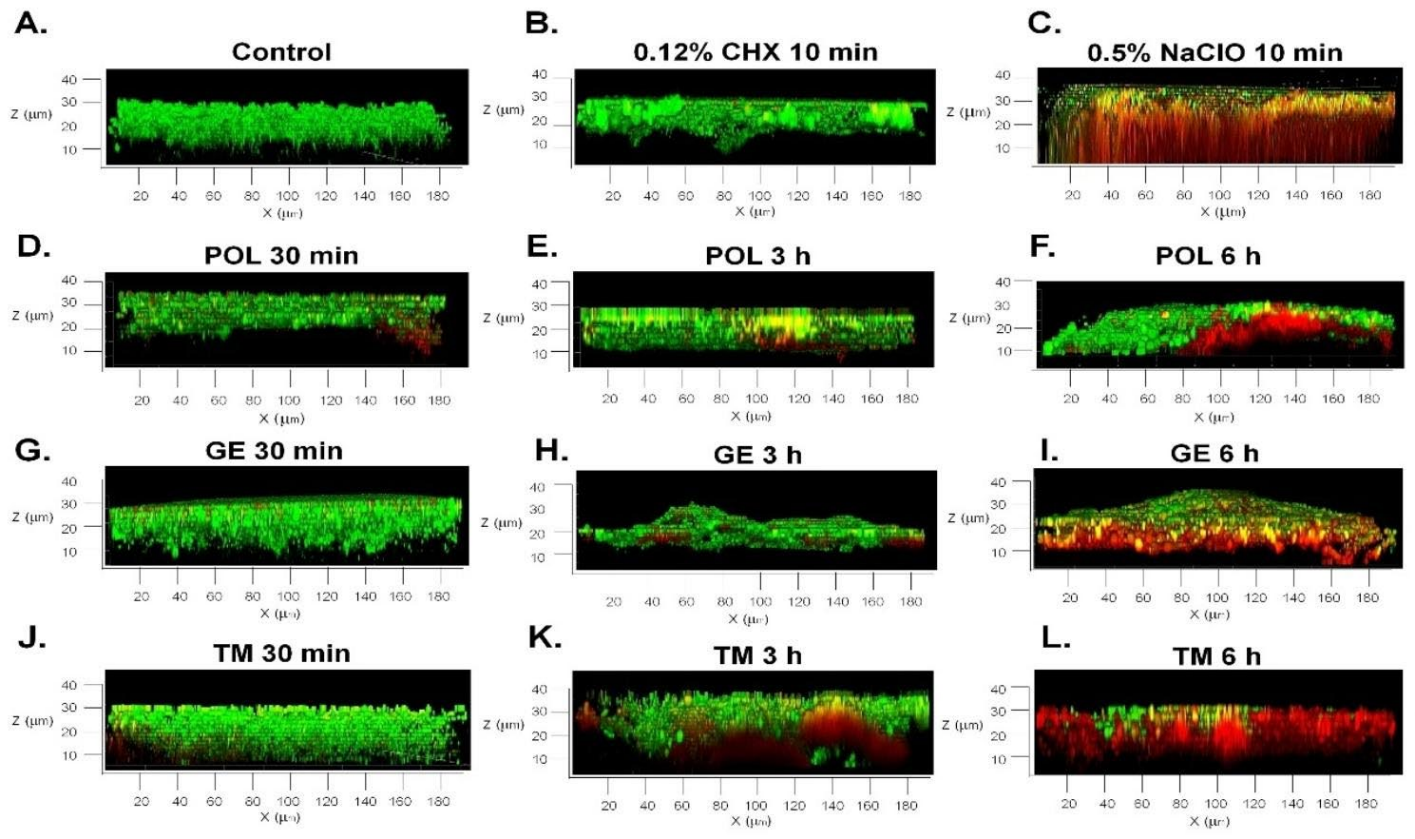
Lateral CLSM view (XZ section) of multispecies biofilms formed on denture acrylics and grown for 72 h. The specimens were immersed in medium without treatment **(A)** and in 5 different tested cleansers for 30 min and 3 and 6 h: CHX, 20 min **(B)**; NaClO, 10 min **(C)**; and POL **(D-F)**, GE **(G-I)**, and TM **(J-L)**.

The lateral view (Z-axis) of the CLSM images used to observe the 3D biofilm structure (Fig. 4) revealed that the viability of the microbes at the bottom of the biofilm attached to surface of the specimens was reduced. This result showed that the solutions could penetrate the biofilm matrix and kill the microbes inside the biofilms. Moreover, increasing the immersion time enhanced the accumulation of substances within biofilms and promoted the killing activity of the denture cleansing agents.

### Evaluation of the cytotoxicity of the denture cleansing solutions chemical residues

**Fig. 5:**
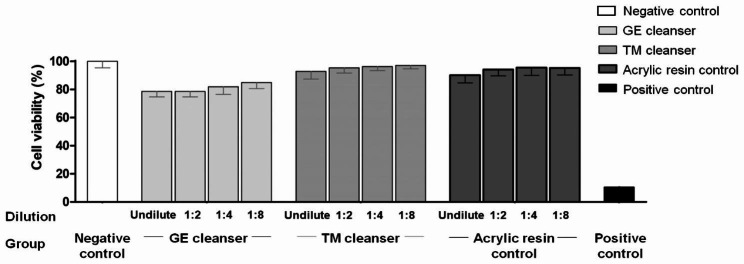
Viability of mouse fibroblast (L929) cells exposed to the GE and TM cleansers impregnated into the acrylic resins for 6 months of simulated daily immersion. The results are expressed as the mean and SD of the percentage of cell viability relative to the negative control without treatment and acrylic resin control

The viabilities of fibroblast (L929) cells exposed to the substances released by the resins (extracts) were evaluated by MTT assay (Fig. 5). The results showed that the extracts from resins immersed in the GE and TM cleansers for 6 months of simulated daily cleansing and the untreated acrylic resin control had no toxic effect on L929 cells (cell viability > 70%). However, the positive control (extracts from 0.1% ZDEC polyurethane film) exhibited a high level of cytotoxicity.

### Determination of the stain removal properties

The colour change of artificial teeth after staining and immersion in cleansers at different lengths of time are presented in Table 1. There was a significant difference between the stain removal efficacies of the cleansing agents and distilled water on artificial teeth stained with tea and coffee after 3 and 6-months of cleaner usage (*p* < 0.001). After 6 months of the staining and cleaning procedures, all of the artificial teeth exposed to tea and immersed in distilled water showed clinically unacceptable colour changes (ΔE > 5.5) and those stained with coffee and immersed in distilled water exhibited noticeable colour changes (ΔE > 4.4). Immersion in 0.5% NaClO, GE, TM and POL solutions resulted in significantly lower ΔE values after tea staining than the control group (distilled water) at 6 months (*p* < 0.05) (Table S3). For the coffee stains, immersion in 0.5% NaClO showed significantly lower ΔE values compared to the control group at 6 months (*p* < 0.01). NaClO had the highest efficacy for removing tea and coffee stains from the acrylic resins. Notably, the 0.12% CHX solution enhanced staining from tea and coffee, and the teeth immersed in this solution showed clinically unacceptable colour changes (ΔE > 5.5) after 3 and 6 months of immersion.

There were no significant differences between the mean ΔE values of the POL, GE and TM denture cleanser groups in term of both tea and coffee stains at all time points (Tables S3 and 4). When the ΔE values were compared according at different time points, the specimens exposed to tea and coffee solutions and cleaned with distilled water (control group) and 0.12% CHX showed significantly increased ΔE values with time (*p* < 0.05).

**Table 1:** Means and standard deviations (SDs) of colour alteration (ΔE) values on artificial teeth after staining and immersion in denture cleansers for 3 and 6 months

Staining solutions	Denture cleansers	ΔE measurement								
		After 1 cycle of staining (1 week)			After 12 cycles of staining and treatment (3 months)			After 24 cycles of staining and treatment (6 months)		
		Mean (min-max)	SD	*P*-value	Mean (min-max)	SD	*P*-value	Mean (min-max)	SD	*P*-value
**Tea**	0.12% CHX	3.05 (1.08–5.97)	1.39	*p* = 0.095	42.99 (40.14–45.06)	1.33	*p* < 0.001	68.09 (64.96–69.86)	1.52	*p* < 0.001
	0.5% NaClO	3.53 (2.12–6.54)	1.41		2.78 (0.41–5.01)	1.67		3.66 (2.15–4.42)	0.78	
	Polident®	4.44 (2.90–6.17)	0.98		4.79 (3.80–6.39)	0.83		4.15 (2.77–5.67)	0.99	
	GE solution	3.29 (2.66–4.24)	0.53		4.02 (2.48–5.95)	1.04		4.11 (1.86–6.84)	1.30	
	TM solution	3.22 (1.83–4.68)	1.04		4.49 (2.92–5.62)	0.93		4.13 (2.64–5.32)	0.97	
	Distilled water	3.60 (2.93–4.48)	0.55		5.19 (4.18–7.17)	1.08		5.95 (4.77–7.34)	0.88	
**Coffee**	0.12% CHX	3.95 (3.14–4.66)	0.56	*p* = 0.075	5.55 (4.55–6.98)	0.77	*p* < 0.001	13.61 (10.73–17.03)	2.38	*p* < 0.001
	0.5% NaClO	3.17 (1.14–4.63)	1.27		3.44 (1.63–4.56)	0.97		2.24 (0.36–3.60)	1.02	
	Polident®	2.97 (1.92–3.68)	0.49		3.23 (2.43–3.69)	0.37		3.63 (3.04–4.69)	0.53	
	GE solution	3.18 (1.93–4.64)	0.92		4.01 (2.71–4.51)	0.56		3.18 (1.61–4.66)	0.86	
	TM solution	3.08 (2.55–3.66)	0.34		3.64 (2.32–5.03)	0.93		3.66 (3.06–4.23)	0.42	
	Distilled water	3.09 (2.62–3.42)	2.57		3.34 (2.32–3.88)	0.47		4.89 (4.10–7.52)	1.02	

### Colour stability and surface roughness of acrylic resins after exposure to denture cleansers

One-way ANOVA revealed significant differences in acrylic resin surface roughness (ΔRa) and colour change (ΔE) among the experimental groups (Table 2). The 0.5% NaClO solution increased the surface roughness approximately twofold compared to the POL, GE and TM groups (*p* < 0.05) and did not exhibit a significant difference compared to the water and 0.12% CHX groups. Furthermore, immersion in 0.5% NaClO caused a significantly greater colour change than the other groups (*p* < 0.01). No significant differences in the mean ΔRa and ΔE values of acrylics after 6 months of simulated daily immersion was found among the POL, GE and TM treatments or the distilled water control group (Table 2).

**Table 2:** Differences in surface roughness (ΔRa) and colour (ΔE) values of the acrylic resins between the baseline and after immersion in the test solutions for 6 months of daily cleansing (T180)

Experimental Groups	Mean ΔRa ± SD	Mean ΔE ± SD
Distilled water	0.012 ± 0.004	0.915 ± 0.163^b^
0.12% CHX	0.012 ± 0.004	0.704 ± 0.261^b^
0.5% NaClO	0.015 ± 0.122	2.249 ± 0.694^a^
Polident®	0.008 ± 0.004*	0.718 ± 0.152^b^
GE solution	0.009 ± 0.005*	1.110 ± 0.263^b^
TM solution	0.007 ± 0.003*	0.805 ± 0.227^b^
*P-*value	0.003	< 0.001

* Indicates a significant difference compared with 0.5% NaClO (*p* value < 0.05). Different superscript letters indicate significant differences at *p* < 0.05

## Discussion

Since denture cleanliness and hygiene are important and necessary, the commercial denture cleansing products market has been growing continuously. Among these products, chemical cleansers, especially tablets and solutions, are simple, comfortable and nonabrasive. In the present study, the effects of two novel denture cleansers with natural active agents, geraniol and thymol were evaluated against multispecies microbial biofilms and in terms of stain removal efficacy and denture surface characteristics and compared to common available cleansing agents: 0.5% NaClO, Polident® and 0.12% CHX. Denture specimens for studying biofilm formation and mechanical properties were prepared from heat-cured polymethyl methacrylate (PMMA) because it is the most widely used material for denture applications and its porous surface favours the accumulation of dental plaque [27].

Among the available denture cleansers, sodium hypochlorite (0.5% and 1% NaClO) is one of the most commonly and widely used disinfectants [28, 29] and has both bactericidal and fungicidal properties. Its microbial effects originate from the hydroxide (OH.) and chloride (Cl^−^) ions that mediate microbial cell wall damage and degrade lipids and fatty acids. However, this solution has several disadvantages, such as causing corrosion of the metal components of the prostheses, increasing the surface roughness of acrylic resins, and having a strong odour and bad taste [30]. Chlorhexidine gluconate is one of the most widely used antiseptic agents in dentistry. It is an important component of oral rinses, mouthwashes and denture cleansers [19] due to its antibacterial and antifungal effects. It is a positively-charged molecule that binds to negatively charged sites of the bacterial cell wall to disrupt the cell wall. Polident® is a peroxide-based effervescent denture tablet that produces oxygen bubbles after coming in contact with water, resulting in mechanical cleaning of the denture surfaces. It also contains sodium lauryl sulfate, which act as a surfactant. Studies [31, 32] have demonstrated that Polident® has antimicrobial effects, biofilm inhibition and stain-removing effects.

Two novel denture cleansing agents in this study contain geraniol and thymol which exhibiting antimicrobial and antibiofilm effects [33–35]. Geraniol (an acyclic monoterpene) and thymol (a monocyclic monoterpene) are common constituents of several essential oils. Their biological activities include making microbial cell membranes more permeable and binding to essential intracellular sites to destroy their structures.

Denture plaque or biofilm is defined as a dense layer of microbial communities embedded in a polymeric matrix that formed on the surface of denture appliances [36]. Our study evaluated the effects of all cleansers on multispecies biofilms including *S. sanguinis, S. mutans, S. aureus, E. coli* and *C. albicans* that formed on saliva-coated acrylic strips to mimic the conditions in the oral cavity. Oral streptococci such as *S. sanguinis* and *S. mutans* are considered the pioneer colonizers during dental plaque formation [37], and these bacteria can bind to salivary pellicles via adhesins. Recent studies have demonstrated that *S. aureus* can incorporate into biofilms formed by oral streptococci [38]. Furthermore, *S. aureus* can trigger the conversion of a homeostatic biofilm into a dysbiotic biofilm and contributes to the development of oral diseases such as staphylococcal mucositis [39]. *Candida* spp. interacts with *Streptococcus* spp. and *Staphylococcus* spp., to form complex and mixed biofilms. An increase in the presence of *Candida* spp. in denture biofilms is an essential factor in inducing inflammation of the mucosa under the denture base and results in the development of denture stomatitis [40]. Therefore, the polymicrobial biofilm model containing both opportunistic oral bacteria and *Candida* spp. was used.

The removal of biofilms formed on denture resins is a significant feature of denture cleansers. It is well accepted that microbial cells growing in biofilms are much more resistant to antimicrobial agents than their planktonic forms. It was supported by the present study, which demonstrated that it required a shorter exposure period to kill all tested planktonic bacteria and *Candida* compared to those in biofilm form. Our study demonstrated that immersion for more than 3 h, the cleansers Polident®, GE and TM could efficiently penetrate and inhibit multispecies denture biofilms with effects similar to 10 min of immersion in 0.5% NaClO. Moreover, the findings showed that increasing the immersion time enabled the substances to efficiently diffuse into and accumulate in the microbial biofilm and led to an increased antibiofilm effect. In contrast, 20 min of immersion in 0.12% CHX had a less effective antibiofilm effect due to the low penetration of CHX into the mature multispecies biofilm (Figs. 3 and 4). Therefore, increasing the immersion time in 0.12% CHX might enhance the antibiofilm effect and the diffusion of this material into biofilms. As shown by the CLSM analysis results, the cleansing agents could effectively kill bacteria growing in biofilms; however, not all of the cleansers could eliminate all of the bacterial and candida cells. This finding is supported by the results of a previous study [41] which demonstrated that immersion in alkaline peroxides for 15, 30 and 60 min was insufficient to completely kill *C. albicans.* In vitro and in vivo studies [42, 43] showed that combination with brushing and chemical denture cleansing method was more effective against polymicrobial denture biofilm than the brushing alone.

The present study also evaluated the toxicity of the GE and TM residues that remained and impregnated into denture acrylic resin after 6 months of simulated daily immersion. LDH and MTT assay were commonly used colorimetric cytotoxicity methods for medical devices and extracts of a device [44]. The LDH assay was used to assess cell death based on measurement lactate dehydrogenase level released from damaged cells. While, the MTT assay determined cell viability by assessing the functional status of mitochondria. The MTT assay was used in this study because it was a simple, sensitive and reproducible method to evaluate cell viability and also the toxicity of dental materials and denture cleansers [25, 45]. Furthermore, this study used the L929 mouse fibroblasts, a standard cell line recommended by the international standards (ISO-10993-5). However, the oral soft tissues including mucosal epithelium and gingival epithelium might come into contact with denture cleanser residues. Therefore, further investigation is warranted to assess the cytotoxicity of cleanser residues on oral epithelial cells and gingival keratinocytes.

The removal of stains deposited on the surfaces of dentures and artificial teeth is an important characteristic of denture cleansers. The stains caused the colour to be different between natural and artificial teeth, which affects the esthetics and longevity of the prosthesis. The present study used the CIE L*a*b* colour system to measure colour difference (ΔE). The threshold of colour difference values was based on Douglas et al. [24], were ΔE < 2.6 was taken as perceptible and ΔE > 5.5 was considered clinically unacceptable. Artificial teeth were incubated in artificial saliva before staining to increase the uptake of stains. Our study simulated 6 months of staining with tea and coffee followed by a 1-hour daily immersion in one of 5 different cleansers, and we found that the 0.12% CHX solution caused the most discolouration of artificial teeth with clinically unacceptable results (ΔE > 5.5). This finding supported previous studies [19, 46] that chlorhexidine gluconate promoted heavy discolourations of artificial teeth. Even though, our study used a low concentration of CHX, it still enhanced dentures staining. The CHX solution with concentrations lower than 0.1% are generally not recommended because its antiplaque efficacy remains unclear [47]. Thus, individuals should be aware of this fact when cleaning with CHX by long-term immersion and everyday use. The findings demonstrated that immersion in 0.5% NaClO caused a smaller difference in colour of the artificial teeth between before and after staining with tea and coffee than the other cleansers. Sodium hypochlorite is widely used as a bleaching agent with the effects of chlorine, a strong oxidizing agent. In vitro and in vivo studies [48, 49] have reported that the NaClO efficiently removes stains on discoloured dentures. The stain removal capacity of the GE and TM cleansers results from the actions of sodium lauryl sulfate (SLS, known as a surfactant) and sodium hexametaphosphate (a tooth whitening agent). The cleaning capabilities of the GE and TM cleansers against tea- and coffee-stained dentures were not significantly different from the commercially available Polident®.

The physical properties of acrylic resins after immersion in denture solutions simulating of 6 months of daily cleaning were also evaluated. Immersion in 0.5% NaClO significantly increased the roughness of the acrylic resins compared to POL, GE and TM. This result was consistent with a previous study [50] showing that 20 min of 0.5% NaClO immersion continued for 21 days promoted a significant change in the resin surface roughness; moreover, increased roughness was associated with a higher NaClO concentration. Changes to the resin surface result from the ability of NaClO to oxidize organic compounds. Based on the National Bureau of Standards (NBS) units for evaluating the colour differences of resins, the NBS unit values were calculated by ΔE × 0.92. NaClO immersion caused a significantly greater colour change (ΔE = 2.249; NBS = 2.069) than the other cleansers. However, the colour changes observed with all cleansers were within the clinically accepted range for acrylic colour differences (NBS unit < 3.0) [51].

This study had some limitations. It is important to emphasize that the present study evaluated the antibiofilm and stain removal efficacy of the chemical agents in vitro. Therefore, certain conditions of the oral environment, such as the composition and pH of saliva, were not simulated. Furthermore, 72 h formed biofilm used to evaluate antibiofilm effect does not fully simulate in vivo denture plaque. The time period of the study was six months of daily cleansing, and denture cleansers may be used for a much longer time. In addition, this study employed a single cell line (L929) to assess the cytotoxicity of the cleanser residues, and future studies should explore other cell lineages, including oral epithelium and keratinocytes.

## Conclusion

Two novel denture cleansing agents containing natural products, geraniol and thymol possess ideal properties for denture cleansers, and exhibited effective antimicrobial, antibiofilm and stain removal capabilities without disturbing the physical properties of the acrylics or causing toxicity due to residual substances extracted from the resins.

### Electronic supplementary material

Below is the link to the electronic supplementary material.

Supplementary Material 1

## Data Availability

The datasets used and analyzed during the current study are available from the corresponding author on reasonable request.

## List of abbreviations

NaClO: sodium hypochlorite
CHX: Chlorhexidine
GE: Geraniol
TM: Thymol
POL: Polident®
CLSM: Confocal Laser Scanning Microscope
NBS: National Bureau of Standards
