# Supplementary material for: Effects of two novel denture cleansers on multispecies microbial biofilms, stain removal and the denture surface: an in vitro study
Source: BMC Oral Health. 2023 Nov 11;23:852. doi: 10.1186/s12903-023-03535-5 (PMC10640750; doi:10.1186/s12903-023-03535-5)
Supplement: Supplementary file 1 — Supplementary Material 1 [file 12903_2023_3535_MOESM1_ESM.docx]

**Table S1** Solutions used in this study

| **Solutions** | **Form** | **Manufacturer** | **Compositions** | **Immersion time/cycle** |
| --- | --- | --- | --- | --- |
| 1. 0.5% NaClO | liquid | MDent, Faculty of Dentistry, Mahidol University | Dilution from 2.5% (v/v) Sodium hypochlorite | 10 min |
| 2. 0.12% (CHX) Chlorhexidine gluconate | liquid | MDent, Faculty of Dentistry, Mahidol University | 0.12% (v/v) Chlorhexidine gluconate | 20 min |
| 3. Polident^®^ (POL) | Tablet | Stafford-Miller Limited, Ireland | Potassium Monopersulfate, Sodium carbonate, Sodium benzoate, Sodium bicarbonate, Citric acid, PEG-180, Sodium Lauryl Sulfate, Tetraacetylethylenediamine | 15 min |
| 4. Geraniol (GE) solution | Tablet | This study | Sodium Bicarbonate, Citric acid, Tartaric acid, Geraniol, Ethylene diamine tetra-acetic acid, Sodium Lauryl Sulfate, Sodium hexametaphosphate, Mannitol | 3 hours |
| 5. Thymol (TM) solution | Tablet | This study | Sodium Bicarbonate, Citric acid, Tartaric acid, Thymol, Ethylene diamine tetra-acetic acid, Sodium Lauryl Sulfate, Sodium hexametaphosphate, Mannitol | 3 hours |

**Table S2** Comparison of antibiofilm efficacy between different chemical methods using one way ANOVA

| **Substances** | **Medium (control)** | **CHX 20 min** | **NaClO 10 min** | **POL 30 min** | **POL 3 h** | **POL 6 h** | **GE 30 min** | **GE 3 h** | **GE 6 h** | **TM 30 min** | **TM 3 h** |
| --- | --- | --- | --- | --- | --- | --- | --- | --- | --- | --- | --- |
| **CHX_20 min** | **0.010** |  |  |  |  |  |  |  |  |  |  |
| **NaClO_10 min** | **<0.001** | **<0.001** |  |  |  |  |  |  |  |  |  |
| **POL_30 min** | **<0.001** | **0.017** | **<0.001** |  |  |  |  |  |  |  |  |
| **POL_3 h** | **<0.001** | **<0.001** | 0.458 | **0.029** |  |  |  |  |  |  |  |
| **POL_6 h** | **<0.001** | **<0.001** | 0.458 | **0.029** | 1.000 |  |  |  |  |  |  |
| **GE_30 min** | **<0.001** | **<0.001** | 0.161 | 0.127 | 1.000 | 1.000 |  |  |  |  |  |
| **GE_3 h** | **<0.001** | **<0.001** | 1.000 | **<0.001** | 0.475 | 0.475 | 0.170 |  |  |  |  |
| **GE_6 h** | **<0.001** | **<0.001** | 0.690 | **<0.001** | **0.003** | **0.003** | **0.004** | 0.673 |  |  |  |
| **TM_30 min** | **<0.001** | **<0.001** | 0.208 | 0.095 | 1.000 | 1.000 | 1.000 | 0.219 | **0.006** |  |  |
| **TM_3 h** | **<0.001** | **<0.001** | 1.000 | **<0.001** | 1.000 | 1.000 | 0.732 | 1.000 | 0.139 | 0.803 |  |
| **TM_6 h** | **<0.001** | **<0.001** | 0.110 | **<0.001** | **<0.001** | **<0.001** | **<0.001** | 0.104 | 1.000 | **<0.001** | **0.007** |

**Table S3** Comparison of tea stain removal efficacy at 3 and 6 months of immersions between different chemical methods using one way ANOVA

| **Substances for 3 months** | **Distilled water** | **0.12% CHX** | **0.5% NaClO** | **GE solution** | **TM solution** |
| --- | --- | --- | --- | --- | --- |
| **0.12% CHX** | **<0.001** |  |  |  |  |
| **0.5% NaClO** | **0.001** | **<0.001** |  |  |  |
| **GE solution** | 0.311 | **<0.001** | 0.239 |  |  |
| **TM solution** | 0.805 | **<0.001** | **0.039** | 0.961 |  |
| **Polident**^®^ | 0.979 | **<0.001** | **0.009** | 0.744 | 0.994 |

| **Substances for 6 months** | **Distilled water** | **0.12% CHX** | **0.5% NaClO** | **GE solution** | **TM solution** |
| --- | --- | --- | --- | --- | --- |
| **0.12% CHX** | **<0.001** |  |  |  |  |
| **0.5% NaClO** | **0.001** | **<0.001** |  |  |  |
| **GE solution** | **0.018** | **<0.001** | 0.955 |  |  |
| **TM solution** | **0.020** | **<0.001** | 0.945 | 1.000 |  |
| **Polident**^®^ | **0.022** | **<0.001** | 0.935 | 1.000 | 1.000 |

**Table S4** Comparison of coffee stain removal efficacy at 3 and 6 months of immersions between different chemical methods using one way ANOVA

| **Substances for 3 months** | **Distilled water** | **0.12% CHX** | **0.5% NaClO** | **GE solution** | **TM solution** |
| --- | --- | --- | --- | --- | --- |
| **0.12% CHX** | **<0.001** |  |  |  |  |
| **0.5% NaClO** | 1.000 | **<0.001** |  |  |  |
| **GE solution** | 0.374 | **<0.001** | 0.554 |  |  |
| **TM solution** | 0.950 | **<0.001** | 0.992 | 0.881 |  |
| **Polident**^®^ | 1.000 | **<0.001** | 0.988 | 0.209 | 0.826 |

| **Substances for 6 months** | **Distilled water** | **0.12% CHX** | **0.5% NaClO** | **GE solution** | **TM solution** |
| --- | --- | --- | --- | --- | --- |
| **0.12% CHX** | **<0.001** |  |  |  |  |
| **0.5% NaClO** | **<0.001** | **<0.001** |  |  |  |
| **GE solution** | 0.050 | **<0.001** | 0.576 |  |  |
| **TM solution** | 0.282 | **<0.001** | 0.155 | 0.961 |  |
| **Polident**^®^ | 0.260 | **<0.001** | 0.170 | 0.970 | 1.000 |
